# Supplementary material for: Contrasting Genomic Trajectories of Bartonellaceae Symbionts of Planthoppers
Source: Genome Biol Evol. 2025 Nov 20;17(12):evaf216. doi: 10.1093/gbe/evaf216 (PMC12661939; doi:10.1093/gbe/evaf216)
Supplement: evaf216_Supplementary_Data [file evaf216_supplementary_data.zip › 00_Supplementary_Figures_alt.pdf]

Supplementary Figures

Diverse genomic trajectories of Bartonellaceae symbionts of planthoppers

Mingjie Ma[1,2 *], Anna Michalik[3], Junchen Deng[2,4], Yi Hu1, Piotr Łukasik[2 *]

1. Ministry of Education Key Laboratory for Biodiversity Science and Ecological Engineering, College of Life Sciences, Beijing Normal University, Beijing 100875, China
2. Institute of Environmental Sciences, Faculty of Biology, Jagiellonian University, Kraków, Poland
3. Department of Developmental Biology and Morphology of Invertebrates, Institute of Zoology and Biomedical Research, Faculty of Biology, Jagiellonian University, Kraków, Poland
4. Doctoral School of Exact and Natural Sciences, Jagiellonian University, Kraków, Poland

* to whom correspondence should be addressed. E-mail: mamingjie@mail.bnu.edu.cn; p.lukasik@uj.edu.pl

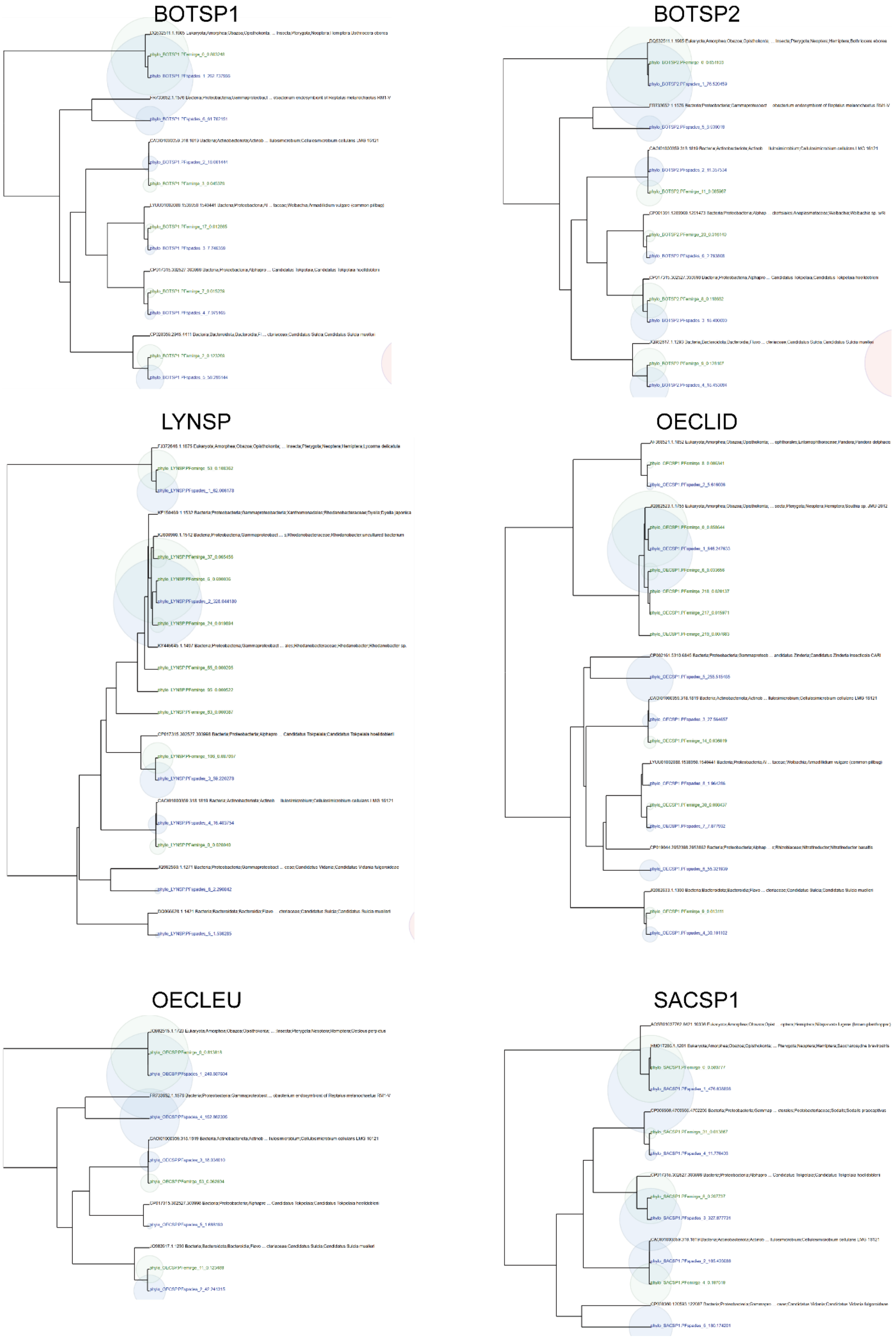

Figure S1. PhyloFlash-based 16S rRNA phylogenetic reconstruction of bacterial community composition in six planthopper samples containing *Bartonellaceae*. Colored labels indicate the source of sequences, with blue showing sequences assembled by SPAdes, green showing sequences reconstructed by EMIRGE. Black labels correspond to the closest-matching reference sequences from the SILVA database. The reconstructed sequences are provided in Table S2

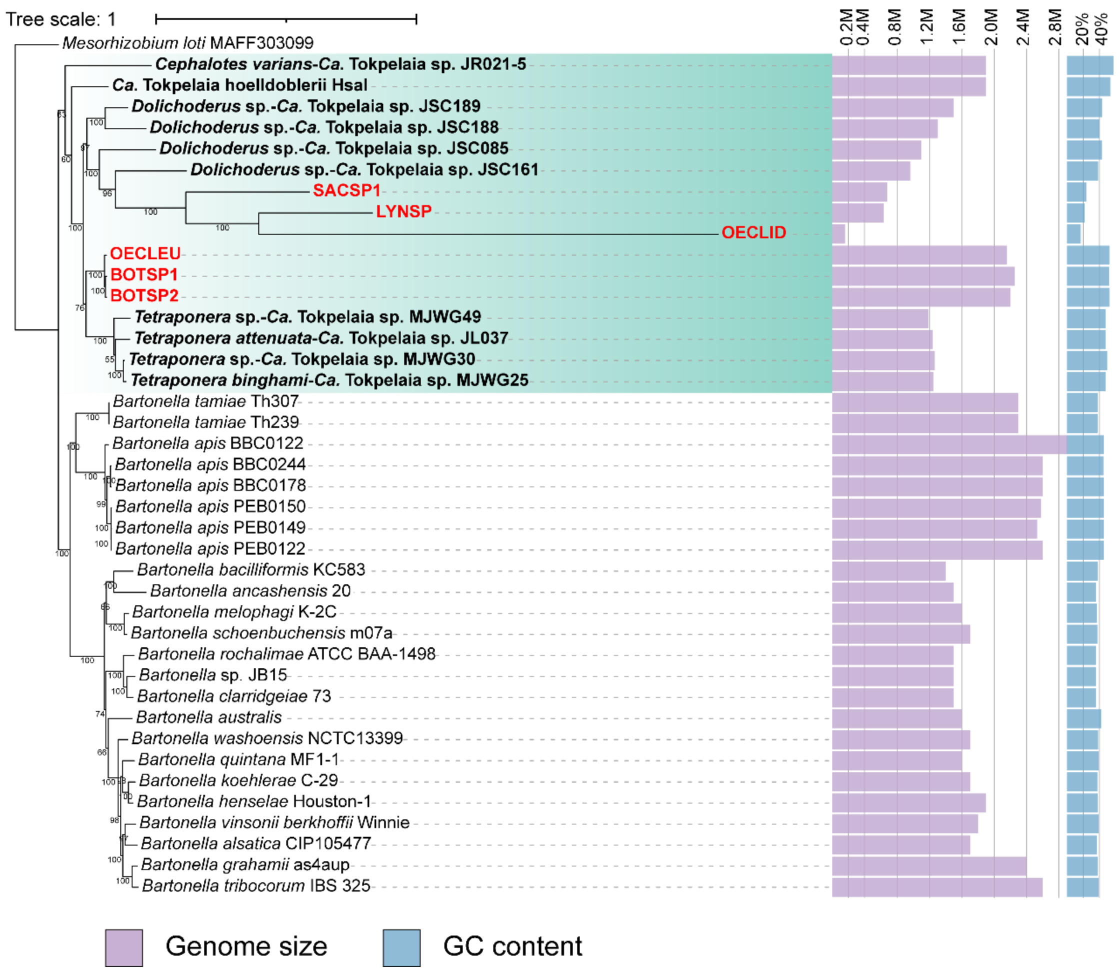

Figure S2. Phylogenetic relationships of *Bartonellaceae* symbionts of planthoppers, ants and other closely related bacteria. Maximum likelihood analysis of *Bartonellaceae* strains with sequenced genomes, based on 68 homologous single-copy genes. The purple bars represent the genome size, and the blue bars - the GC content. The *Tokpelaia* clade is indicated with green boxes; bold black labels indicate ant-associated, and bold red – planthopper-associated strains. Bootstrap support values above 50% are shown by the respective nodes. Unlike in Fig. 2A, OECLID branch has not been truncated.

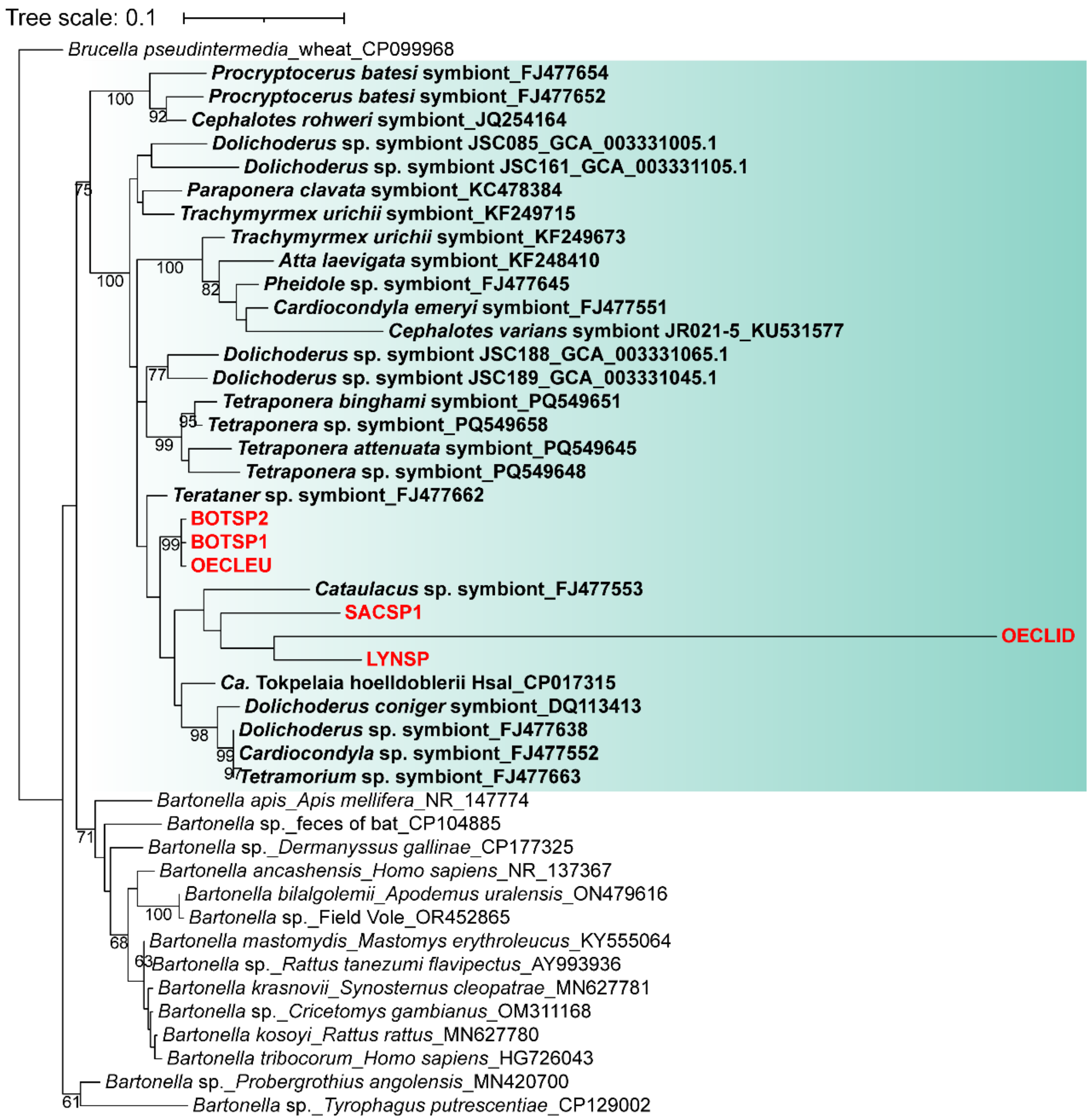

Figure S3. Maximum likelihood phylogeny of full-length 16S rRNA gene sequences of *Bartonellaceae* associated with planthoppers, ants, and selected other hosts, derived from metagenomes and the NCBI database. The *Tokpelaia* clade is indicated with green boxes; bold black labels indicate ant-associated, and bold red – planthopper-associated strains. Bootstrap support values above 50% are shown by the respective nodes. Unlike in Fig. 2B, OECLID branch has not been truncated.

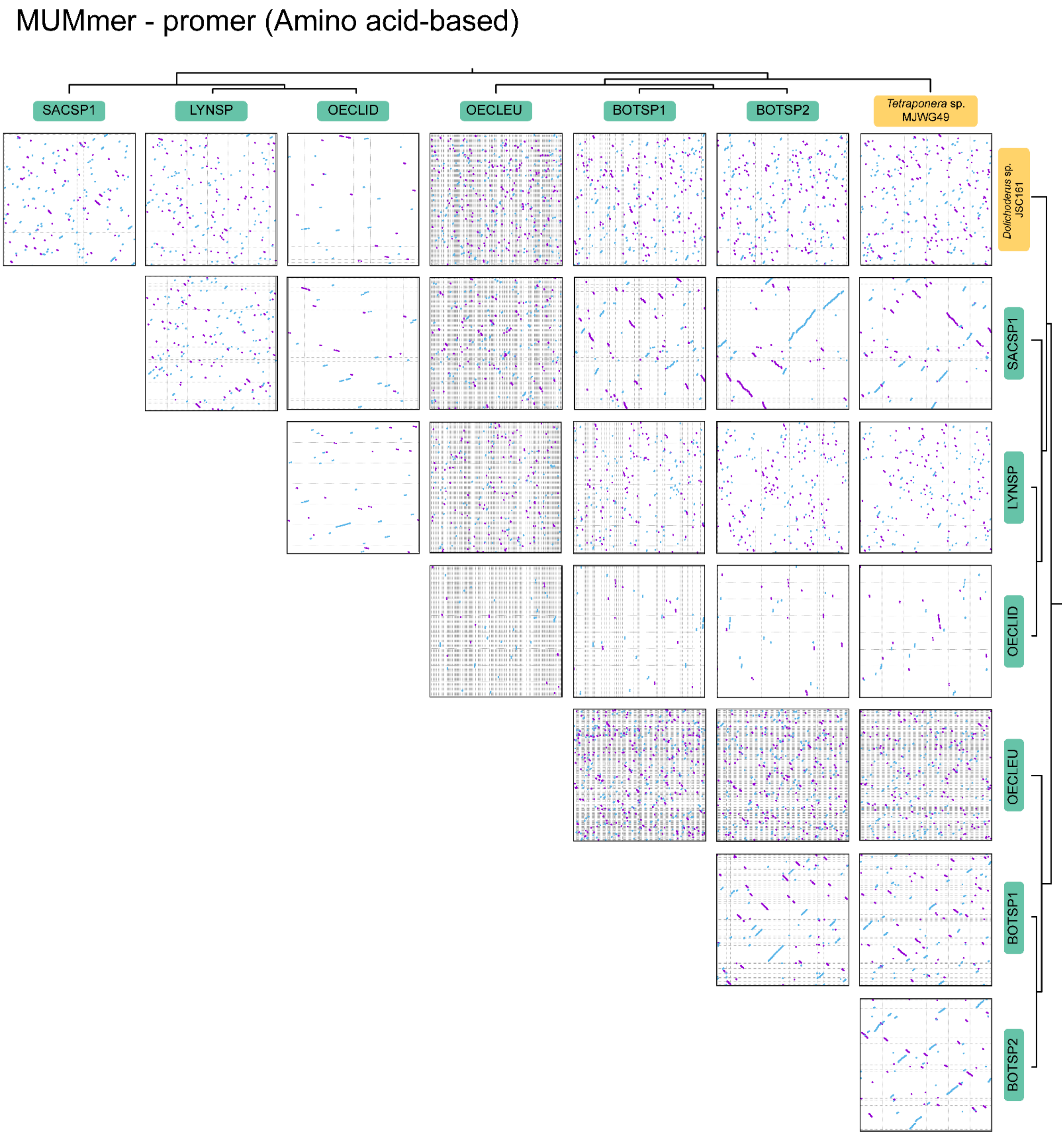

Figure S4. Dot plots of protein-based alignments among different *Tokpelaia* strains. Comparison of gene order in the genome contigs of *Tokpelaia* symbionts associated with planthoppers, and the genomes of *Dolichoderus* and *Tetraponera* ant symbionts. Comparisons have been done using promer v3.07, with default settings; lines and dots represent regions with significant amino acid sequence similarity, with forward matches colored in purple and reverse matches in blue.

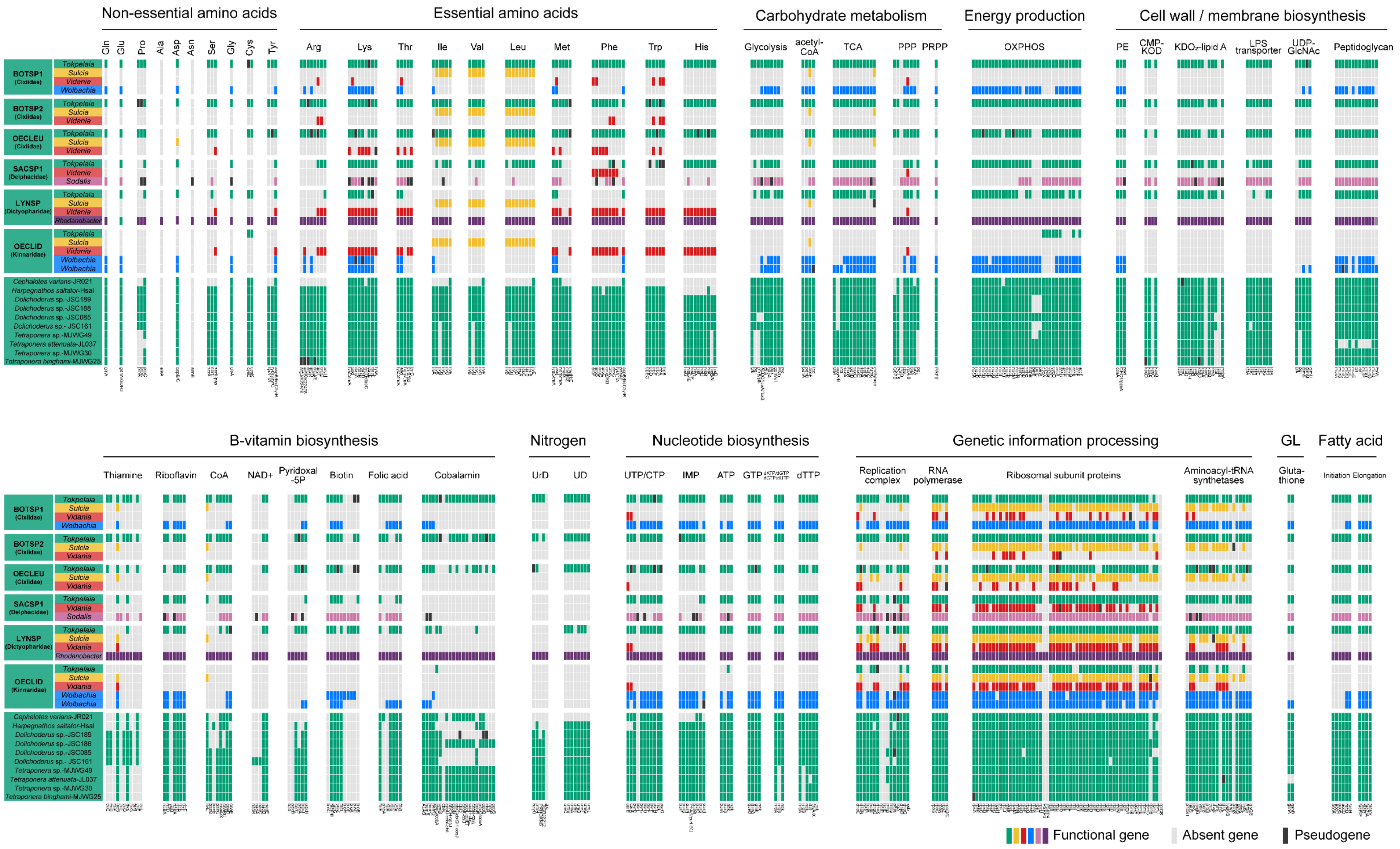

Figure S5. The comparison of gene sets related to the biosynthesis of amino acids, vitamins, cell membrane components, nucleotides, fatty acids, carbohydrate metabolism, nitrogen cycling, energy production, and genetic information processing in multi-partite symbioses of six planthopper species and in *Tokpelaia* of ten ant species. Each bar represents a single gene, with a name abbreviated at the bottom. Genes are classified as functional (colored), absent (light gray), and likely pseudogenes (black).

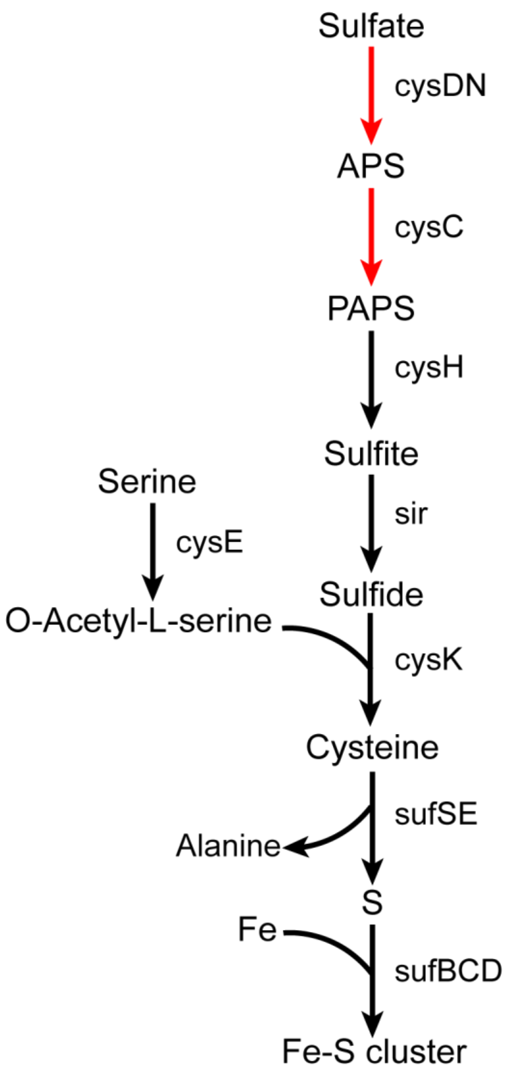

Figure S6. The completeness of the sulfur-related metabolic pathway retained in the genome of the Tokpelaia symbiont of Oeclidius sp. (OECLID). Black and red arrows indicate genes scored as present and absent, respectively.
